# Complete Genome Sequence of Soil Bacterium *Burkholderia* sp. Strain FERM BP-3421, a Producer of Spliceostatins

**DOI:** 10.1128/mra.00111-23

**Published:** 2023-04-19

**Authors:** Sylvia Kunakom, Barbara I. Adaikpoh, Tuan Anh Tran, Alessandra S. Eustáquio

**Affiliations:** a Department of Pharmaceutical Sciences, College of Pharmacy, University of Illinois at Chicago, Chicago, Illinois, USA; b Center for Biomolecular Sciences, College of Pharmacy, University of Illinois at Chicago, Chicago, Illinois, USA; Portland State University

## Abstract

Here, we report the complete genome sequence of *Burkholderia* sp. strain FERM BP-3421, a bacterium isolated previously from a soil sample in Japan. Strain FERM BP-3421 produces spliceostatins, which are splicing modulatory antitumor agents that advanced to preclinical development. The genome is composed of four circular replicons of 3.90, 3.0, 0.59, and 0.24 Mbp.

## ANNOUNCEMENT

Strain FERM BP-3421 is a soil isolate originally assigned as Pseudomonas sp. no. 2663 based on morphological and physiological characteristics (1). It was later revised to *Burkholderia* sp. based on 16S rRNA sequence analysis (accession no. KJ364655; 100% identity to Burkholderia rinojensis) (2). It was obtained from the International Patent Organism Depository at the National Institute of Advanced Industrial Science and Technology, Tsukuba, Japan (accession code FERM BP-3421) and routinely cultured in lysogeny broth (LB) medium at 25 to 30°C.

Strain FERM BP-3421 produces the spliceostatin family of antitumor natural products, such as FR901464, spliceostatin B, and thailanstatin A at a gram per liter scale which facilitated their preclinical development as antibody drug conjugates (2–7). Moreover, this strain represents a promising heterologous host (8, 9). To facilitate host development, we henceforth report its genome sequence.

Strain FERM BP-3421 was grown in 20 mL LB broth at 30°C and 180 rpm for 18 h. DNA isolation from harvested cells (0.2 g of wet weight) was performed using the Qiagen blood and cell culture DNA Midi kit (PN 13343) according to the manufacturer’s instructions. Illumina and Oxford Nanopore sequencing were performed by the sequencing core at the University of Illinois at Chicago. Shotgun sequencing used a Nextera XT DNA library preparation kit (Illumina, San Diego, CA). The library was sequenced on an Illumina NextSeq 500 instrument, employing paired-end reads of 150 bp, yielding 40,203,038 reads. Nanopore sequencing libraries were generated using the Oxford Nanopore genomic library protocol SQK-NSK007, according to the manufacturer’s instructions (Oxford Nanopore Technologies). Sequencing was performed using a FLO-MIN104 (v. R9) flow cell and a MinION instrument. A total of 45,562 reads (*N*_50_, 11,433 bp) were obtained.

*De novo* assembly was performed with Flye (v. 2.9, number of polishing iterations, -i = 2) on the raw Nanopore reads after trimming for adapter sequences with Porechop (v. 2.4) (10). The obtained Nanopore coverages were 38 (chromosome 1), 38 (chromosome 2), 39 (plasmid p1), and 58 (plasmid p2). Trimmed Nanopore reads were used to polish the genome five times with Racon software (v. 1.4.20; score for matching bases, -m = 8; score for mismatching bases, -x = 6) and once with medaka software (v. 1.4.4) (11). Hybrid polishing with Illumina reads was done four times with pilon (v. 1.20.1) (12). The obtained Illumina coverage was 747x. Default parameters were used for all software except where otherwise noted. Genome annotation was performed using the standalone NCBI Prokaryotic Genome Annotation Pipeline (PGAP; v. 2021-11-29.build5742) (13).

The final genome sequence consists of the following four circular replicons of the indicated size and G+C content: chromosome 1 (3.90 Mbp, 67.7%), chromosome 2 (3.0 Mbp, 67.9%), plasmid 1 (p1; 0.59 Mbp, 66.7%), and plasmid 2 (p2; 0.24 Mbp, 62.0%). The output of the Flye assembly was circular replicons that were not rotated. Genome annotation revealed 6,775 genes, including 6,374 protein-coding sequences; 5 operons containing 5S, 16S, and 23S rRNAs; 60 tRNAs; and 322 pseudogenes.

### Data availability.

The raw sequence reads have been submitted to the SRA under accession numbers SRX18227412 (Nanopore) and SRX18227411 (Illumina), BioSample accession number SAMN31673526, and BioProject accession number PRJNA899914. The assembled genome sequence of *Burkholderia* sp. FERM BP-3421 has been deposited at GenBank under accession numbers CP117779, CP117780, CP117781, and CP117782, which are the versions described in this paper.
